# Identification of Biomarkers for Predicting Ovarian Reserve of Primordial Follicle *via* Transcriptomic Analysis

**DOI:** 10.3389/fgene.2022.879974

**Published:** 2022-05-25

**Authors:** Li Liu, Biting Liu, Ke Li, Chunyan Wang, Yan Xie, Ning Luo, Lian Wang, Yaoqi Sun, Wei Huang, Zhongping Cheng, Shupeng Liu

**Affiliations:** ^1^ Department of Obstetrics and Gynecology, Shanghai Tenth People’s Hospital, School of Medicine, Tongji University, Shanghai, China; ^2^ Institute of Gynecological Minimally Invasive Medicine, School of Medicine, Tongji University, Shanghai, China; ^3^ Department of Clinical Laboratory Medicine, Shanghai Tenth People’s Hospital, School of Medicine, Tongji University, Shanghai, China

**Keywords:** ovarian reserve, biomarkers, bioinformatics, assisted reproductive technology, transcriptome

## Abstract

Ovarian reserve (OR) is mainly determined by the number of primordial follicles in the ovary and continuously depleted until ovarian senescence. With the development of assisted reproductive technology such as ovarian tissue cryopreservation and autotransplantation, growing demand has arisen for objective assessment of OR at the histological level. However, no specific biomarkers of OR can be used effectively in clinic nowadays. Herein, bulk RNA-seq datasets of the murine ovary with the biological ovarian age (BOA) dynamic changes and single-cell RNA-seq datasets of follicles at different stages of folliculogenesis were obtained from the GEO database to identify gene signature correlated to the primordial follicle pool. The correlations between gene signature expression and OR were also validated in several comparative OR models. The results showed that genes including *Lhx8, Nobox*, *Sohlh1*, *Tbpl2*, *Stk31*, and *Padi6* were highly correlated to the OR of the primordial follicle pool, suggesting that these genes might be used as biomarkers for predicting OR at the histological level.

## Introduction

Ovarian reserve (OR) is used to describe the ovarian ability to provide viable oocytes, mainly determined by the number of primordial follicles in the ovary, which continuously depleted until ovarian senescence (Moolhuijsen and Visser, 2020; Ruth et al., 2021). Therefore, OR declined along with ovary aging, and the biological ovarian age (BOA) in humans has a lifespan of about 50 years (Alviggi et al., 2009; Moolhuijsen and Visser, 2020; Ruth et al., 2021). Several pathological factors, including chromosomal abnormalities, autoimmune disorders, and iatrogenic injuries, can accelerate the depletion of OR, leading to the diminished ovarian reserve (DOR) (Steiner et al., 2017; Spears et al., 2019; Takahashi et al., 2021).

With the development of assisted reproductive technology (ART), such as ovarian tissue cryopreservation (OTC) (Anderson et al., 2017) and transplantation (OTP) (Dolmans et al., 2021), *In vitro* activation (IVA), and growth (IVG) of primordial follicles (Telfer and Andersen, 2021), there is an increasing need for the technical operations directly in ovarian tissue obtained by surgical resection, especially the evaluation of OR at the histological level. In the OTC procedure, for example, ovarian tissue from each patient needs to be processed into several fixed-size ovarian tissue slices (e.g., 0.5 cm*0.5 cm*0.1 cm), whereas the evaluation of OR in ovarian tissue slices is not yet standardized. Traditionally, the gold standard for assessing OR counts the number of primordial follicles on serial H&E/IHC-stained sections slice by slice. However, the time-consuming and highly subjective method makes it inconceivable and ineffective in the clinic (Myers et al., 2004; Youm et al., 2014; Terren et al., 2019; Mahmoudi Asl et al., 2021). Serological hormone testing and ultrasonography are currently used for evaluating OR in the clinic. These methods are based on assessing the quality of the growing follicles but not the number of primordial follicles (Steiner et al., 2017; Lew, 2019). To date, Anti-Müllerian hormone (AMH) is a promising serum marker for assessing OR at the serological level (Visser et al., 2012). However, since AMH is not an oocyte-specific marker, its applicability in histological assessing OR is bound to be limited by many factors, such as the patient’s age and the pathological abnormalities of polycystic ovary syndrome (PCOS) (Dewailly et al., 2014; Moolhuijsen and Visser, 2020). Therefore, an effective and easily used method is still needed for the clinical assessment of OR at the histological level.

Currently, increasing transcriptomic data makes it possible to find out genes specifically expressed in unique types of cells. The present study aimed to identify potential biomarkers for predicting OR by combinational analysis of published transcriptomic data from bulk RNA-seq and single-cell RNA-seq of humans and mice ovarian tissues. We hoped our findings will provide new insights into the clinical evaluation of OR and targeted interventions for fertility preservation.

## Materials and Methods

### Animals and Sample Preparation

The mice were raised in an environment with a temperature of between 18 and 23°C and humidity of between 40 and 60% under 12-h light/dark cycles. Animal experiments were approved by the Animal Ethics Committee of Shanghai Tenth People’s Hospital (No. SHDSYY-2020-Y0688). 2-month-old (*n* = 3) and 8-month-old (*n* = 3) female C57BL/6L mice under specific pathogen-free conditions were provided by Shanghai SLAC Laboratory Animal Corp, Shanghai, China. Bilateral ovariectomy was performed, and ovarian bursas were removed under the microscope according to the previous study (Souza et al., 2019). After three washes with sterile PBS solution, samples were collected from the bilateral ovaries of each mouse for RNA extraction.

### RNA-Seq and Microarray Profiles Acquisition

**FIGURE 1:**
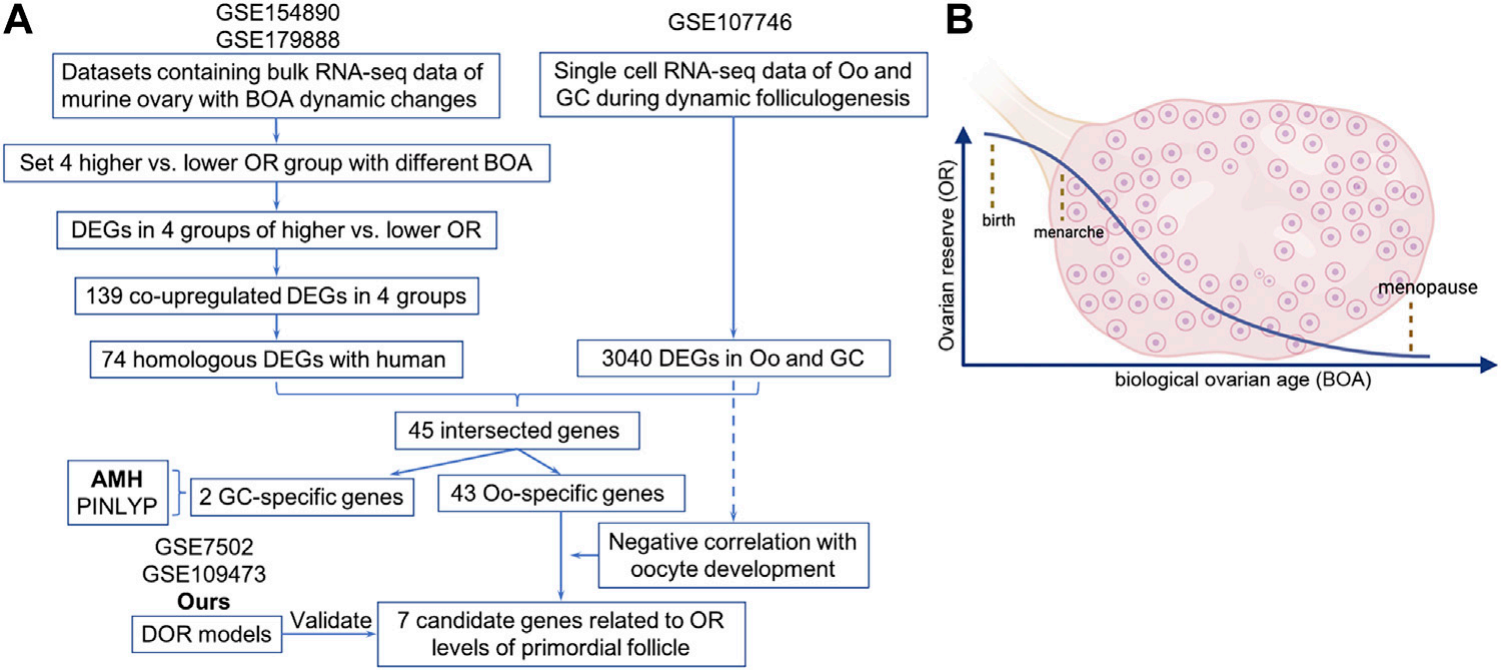
The flowchart of the design and analysis of this study.

Raw files of five registered datasets used in this study were downloaded from NCBI GEO (http://www.ncbi.nlm.nih.gov/geo) (Barrett et al., 2013). The GSE154890 and GSE179888 datasets include bulk RNA-seq data of murine ovary with BOA dynamic changes. The GSE107746 dataset includes single-cell RNA-seq data of human oocytes and granulosa cells of follicles during dynamic folliculogenesis. The GSE7502 and GSE109473 containing microarray profiles of murine ovary were retained for subsequent analyses. For better understanding, the characteristics and processing procedures of datasets were described in Table 1 and the flowchart (Figure 1A).

**TABLE 1:** Characteristics of datasets used in this study.

GSE series	Data type	Platforms	Application	Sample used category
GSE154890	Bulk RNA-seq	GPL16417	Identification	3/6/9/12months
GSE179888	Bulk RNA-seq	GPL17021	Identification	p3/7/14/21/60/y1/y2
GSE107746	scRNA-seq	GPL20795	Identification	ALL
GSE7502	microarray	GPL2552	Validation	O_AL_1/6/16/24 months
GSE109473	microarray	GPL6887	Validation	Lhx8_P7_WT/KO

### Identification of Differentially Expressed Genes

The process of raw data and analysis of differentially expressed genes (DEGs) were performed using the Sangerbox tools, a free online platform for data analysis (http://www.sangerbox.com/tool). DEGs were screened for GSE154890 and GSE179888 datasets with the threshold criterion of *p*-value < 0.05 and log2 |FC| >1. DEGs of GSE107746 were screened for gene expression of log2 (FPKM+1) with the threshold criterion of adj. *p*-value < 0.05 and log2 |FC| >2. Overlapping DEGs were identified using the Venn diagram web tool (https://bioinformatics.psb.ugent.be/webtools/Venn/).

### Analysis of Human and Mouse Homologous Genes

Human and mouse homologous genes were downloaded from the Vertebrate Homology Database (http://www.informatics.jax.org/homology.shtml). Mouse candidate genes were overlapped with human homologous genes to find homologous genes expressed in humans and mice.

### Total RNA Extraction and Quantitative Real-Time PCR

The extraction of the total RNA was completed with RNAiso Plus (TaKaRa, Japan) according to the manufacturer’s instructions. RNA quality and quantity were measured by NanoDrop2000 (Thermo Scientific, Wilmington, DE, United States). Then RNA was reverse-transcribed into complementary DNA using PrimeScript RTMaster Mix (TaKaRa, Japan). Quantitative real-time PCRs (qRT-PCR) were performed on a QuantStudio Dx (ABI, America) using the SYBR Premix ExTaq kit (Takara, Shiga, Japan). The thermal cycler conditions were as follows: 30 s at 95.0°C for cDNA denatured, followed by 40 cycles of 15 s at 95°C and 60°C for the 30 s. Verification of specific product amplification was performed by dissociation curve analysis. The mRNA relative expression was calculated by the 2^-ΔΔCt method with *Gapdh* as an internal control (Livak and Schmittgen, 2001). The experiment was repeated in triplicate, and all primer sequences were listed in Table 2.

**TABLE 2:** Primers used for qPCR validation.

Gene (Mouse)	Forward sequence	Reverse sequence
*Lhx8*	AGC​ACA​GTT​CGC​TCA​GGA​CAA​C	GCT​GAG​GAA​GAA​TGG​TTG​GGA​C
*Nobox*	CGT​TCC​TGG​CAG​TGA​CAG​CAT​A	GGA​ATG​AAC​CCA​ACT​GGC​TGC​T
*Sohlh1*	GCC​AAA​CCA​TCT​GCT​GTG​TCT​C	AAG​GTC​TCT​CCA​GCA​GCT​CTG​A
*Tbpl2*	ACT​CCA​ATG​CCT​TAC​CTG​TGG​C	GCC​AGA​TTT​GCA​GTG​GAA​ACT​AC
*Padi6*	CTG​AGC​GAG​AAG​AGC​AAA​GTG​C	ATG​ACA​CCG​TCT​TGT​GAG​GAG​C
*Stk31*	CGT​GTG​TAG​GAA​CCA​GGC​TGA​A	GGA​CCC​TTC​ATC​CAA​CAC​TTG​C
*Vrtn*	ACC​AAG​AGC​ACC​TTC​TAC​CGC​T	GAA​CTG​CTG​CAA​TGG​CAC​AAA​GC

### Statistical Analysis

Data analysis was performed using SPSS 20.0 statistical software (IBM, New York, NY, United States). Quantitative data were expressed as the standard deviation of the mean. Analysis of quantitative parametric data between two groups was assessed with Student’s t-test. Correlational statistical analysis was completed with the Spearman correlation test in this article. *p* values <0.05 were considered statistically significant.

## Results

### Identification of Differentially Expressed Genes Correlated With Biological Ovarian Age

**FIGURE 2:**
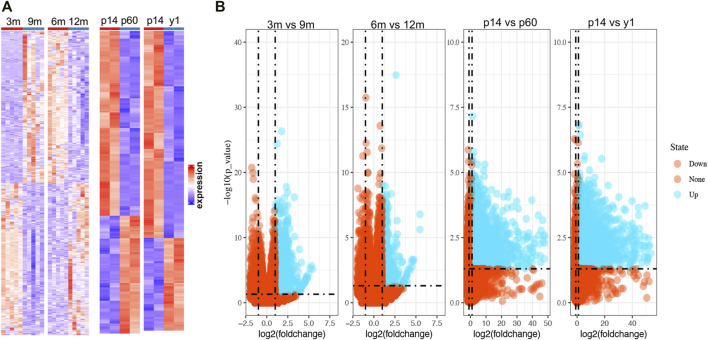
The heatmaps and volcano plots of DEGs in multiple OR comparison groups. **(A)** Heatmaps for DEGs in four OR comparison groups from GSE154890 and GSE179888. The red shading represents the upregulated genes, while the red shading represents the down-regulated genes. **(B)** Volcano plots for DEGs. The blue points represent the upregulated genes, and the red points represent the non-upregulated genes in high OR. DEGs were screened on the criterion of log2|FC| > 1.0 and *p* < 0.05.

Since the natural decline of OR changes and BOA (Figure 1B), genes differentially expressed with BOA were first identified. Two bulk RNA-seq datasets (GSE154890 and GSE179888) containing transcriptome data of mice ovary tissues at different ages were obtained from the GEO database and sent for further differentially expressed genes (DEGs) analysis. Four pairs of higher vs. lower OR groups, namely “3 vs. 9 m”, “6 vs. 12 m”, “p14 vs. p60” and “p14 vs. y1” were respectively conducted simulating young vs. old BOA comparison according to murine age referenced to human as previously reported (Dutta and Sengupta, 2016). DEGs of each group were screened with the threshold criterion of Log2|FC| > 1 and *p* value < 0.05. The cluster analysis showed that ovarian tissues at different ages had unique gene expression profiles (Figure 2A). Multiple DEGs were identified in each group, with 1,640 DEGs in 3 vs. 9 m, 894 DEGs in 6 vs. 12 m, 5,620 DEGs in p14 vs. p60, and 6,485 DEGs in p14 vs. y1, respectively (Figure 2B). The results showed that these DEGs might be involved in murine BOA.

### Screening the Differentially Expressed Genes Related to Human Oocyte

**FIGURE 3:**
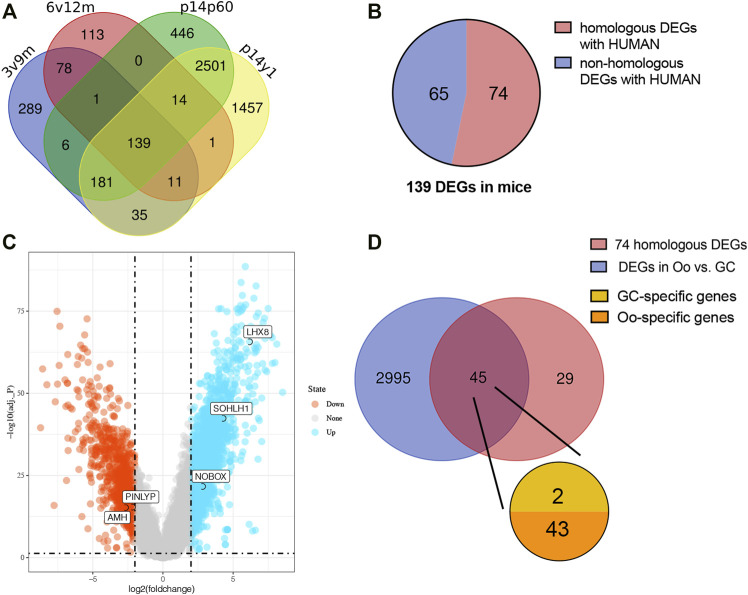
Screening the intersected upregulate DEGs related to human oocyte **(A)** Venn diagram demonstrates the overlap of the murine upregulated DEGs in four higher vs. lower OR groups of BOA comparison. **(B)** The pie chart represents the 139 intersected upregulated DEGs homologous with humans (red) or not (blue). **(C)** Volcano plots for DEGs of human oocytes (Oo) and granulosa cells (GC) from dataset GSE107746. The blue points represent the upregulated genes in Oo, and the red points represent the upregulated genes in GC. DEGs were screened on the criterion of log2|FC| > 2.0 and adj. *p* < 0.05. **(D)** Venn diagram of 74 upregulated murine the homologous DEGs and the DEGs in human Oo and GC (blue) demonstrates the overlap of the upregulated murine DEGs related to human Oo- (orange) or GC-specific (yellow) expression.

Among the DEGs identified above, upregulated DEGs in each younger group were selected for further analysis considering higher OR in the more immature ovaries. To make it more reliable, intersection analysis was firstly performed and found a total of 139 DEGs upregulated in all four sets of groups, including 74 human homologous genes (Figures 3A,B). Considering the uniqueness of follicular structure and folliculogenesis, we hypothesized that potential biomarkers should be expressed explicitly in oocytes. Then the expression of 74 human homologous genes in human oocytes (Oo) and granulosa cells (GC) was investigated using the single-cell sequencing dataset of human Oo and GC (GSE107746). The analysis of the single-cell sequencing dataset identified 3,040 DEGs between human Oo and GC (Figure 3C), including 45 out of 74 human homologous genes identified in murine transcriptomic data (Figure 3D). Among 45 DEGs from both human and murine transcriptomic data, 43 genes were specifically highly expressed in Oo, and two were specifically highly expressed in GC (Figure 3D). It was suggested that 43 Oo specifically expressed genes may be correlated with human OR. Interestingly, our results revealed that the highest fold change gene of these 2 GC-specific genes was *Amh*, the well-known serum marker for OR (Supplymentary Figure S1), validating the feasibility of our screening process.

### Identification of Potential Genes Related to the Follicular Developmental Stages

**FIGURE 4:**
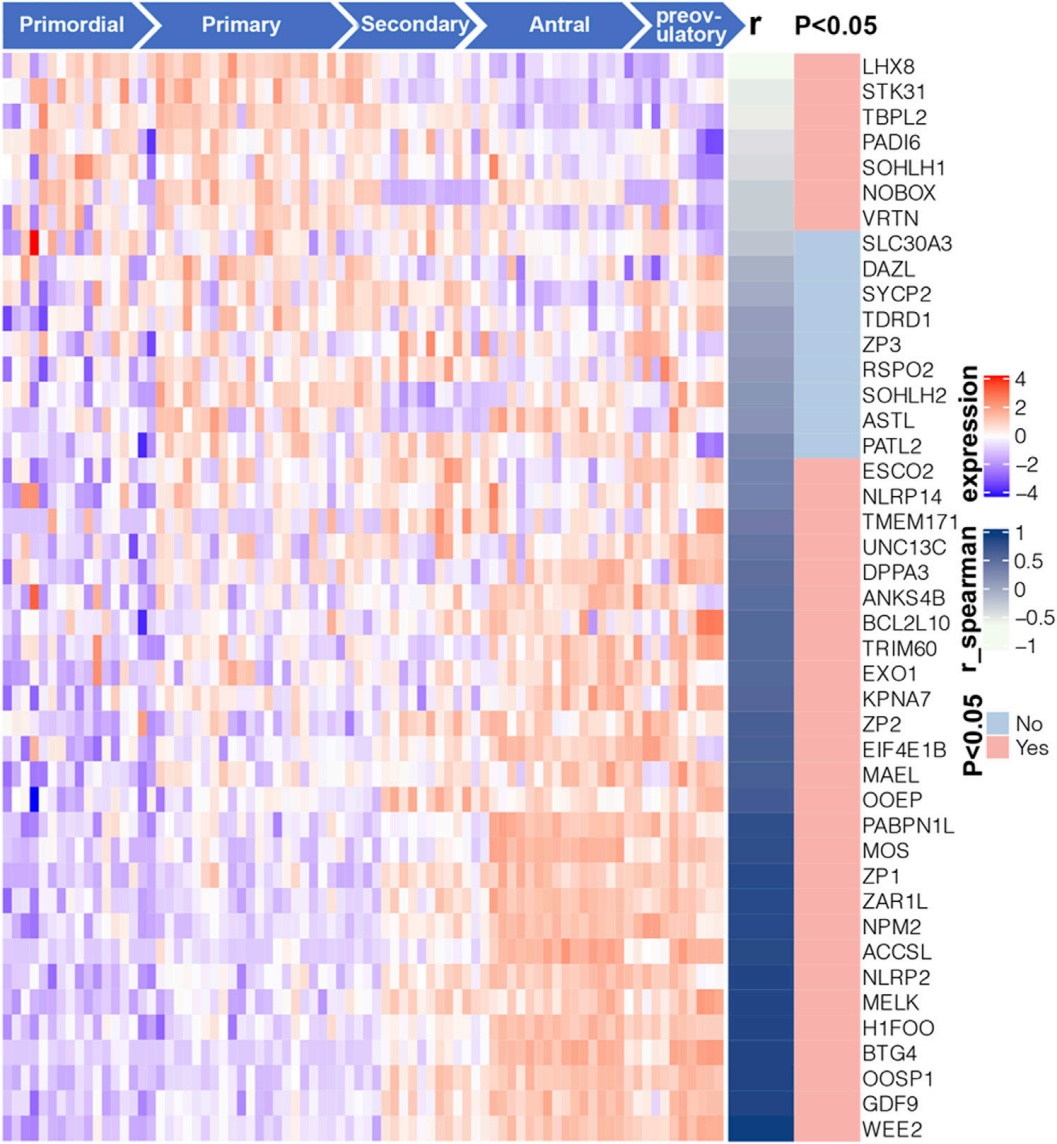
Expression profile and correlation with follicular developmental of 43 candidate Oo-specific genes. Heatmap displays the expression levels of 43 candidate Oo-specific genes in the oocytes at five stages of folliculogenesis in the GSE107746 dataset. The right panel of the heatmap shows the correlation between the expression levels of each gene with the follicular developmental stages in the GSE107746 dataset.

**FIGURE 5:**
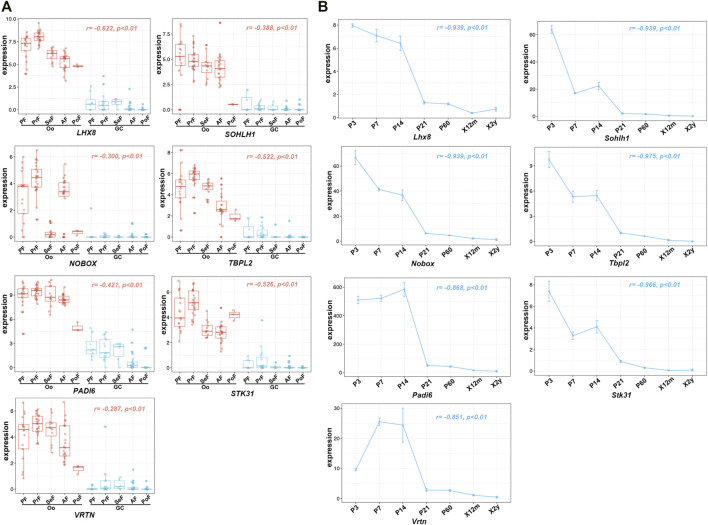
Expression of seven identified potential genes in GSE107746 and GSE179888 datasets. **(A)** The expression levels of seven identified potential genes, *Lhx8*, *Sohlh1*, *Nobox*, *Stk31*, *Tbpl2*, *Padi6*, and *Vrtn* in the human Oo or GC at five stages of folliculogenesis in the GSE107746 dataset. Oo, oocyte; GC, granulosa cells; PF, primordial follicle; PrF, primary follicle; SeF, secondary follicle; AF, antral follicle; PoF, preovulatory follicle; r, correlation coefficient. **(B)** The expression trends with BOA changes in murine ovaries from the GSE179888 dataset. P, postnatal day; X12m, postnatal 12 months; X2y, postnatal 2 years; r, correlation coefficient; p, *p*-value.

To further investigate the relationship of 43 Oo-specific genes during folliculogenesis, these gene expression patterns and follicular developmental stages were evaluated using the single-cell sequencing dataset (GSE107746). The heatmap showed that these Oo-specific candidate genes have different expression patterns during folliculogenesis (Figure 4). As the volume and genetic abundance of oocytes increase with the progressive development of oocytes from primordial follicles to preovulatory follicles, the best potential biomarkers should most likely represent the early stages (primordial and primary stages) during folliculogenesis. Our analysis showed that seven genes, including *Lhx8*, *Sohlh1*, *Nobox*, *Stk31*, *Tbpl2*, *Padi6*, and *Vrtn*, were significantly negatively correlated with the follicular developmental stages (Figure 4). Close investigation showed that these seven genes expressed in Oo declined along with folliculogenesis and remained low in GC (Figure 5A). Moreover, the expression of these genes in the murine ovary at different age were also investigated (GSE179888). It showed that expression of these genes declined significantly and correlated negatively with age (Figure 5B).

In addition, the expression of oocyte-specific genes that were significantly positively correlated with follicular developmental stages was also investigated. Increased expression of these genes was observed in Oo and folliculogenesis and remained low in GC (Supplymentary Figure S2A). In the murine ovary, the expression of these genes was similar to *Amh*, rising to a peak at a one-time point and declining to baseline after that, which was inconsistent with the natural decline of OR (Supplymentary Figure S2B). Taken together, these data showed that *Lhx8*, *Sohlh1*, *Nobox*, *Stk31*, *Tbpl2*, *Padi6*, and *Vrtn* strongly negatively correlated with BOA were potentially biomarkers for evaluating OR at the histological level.

### Validation of the Potential Ovarian Reserve-Related Biomarkers

**FIGURE 6:**
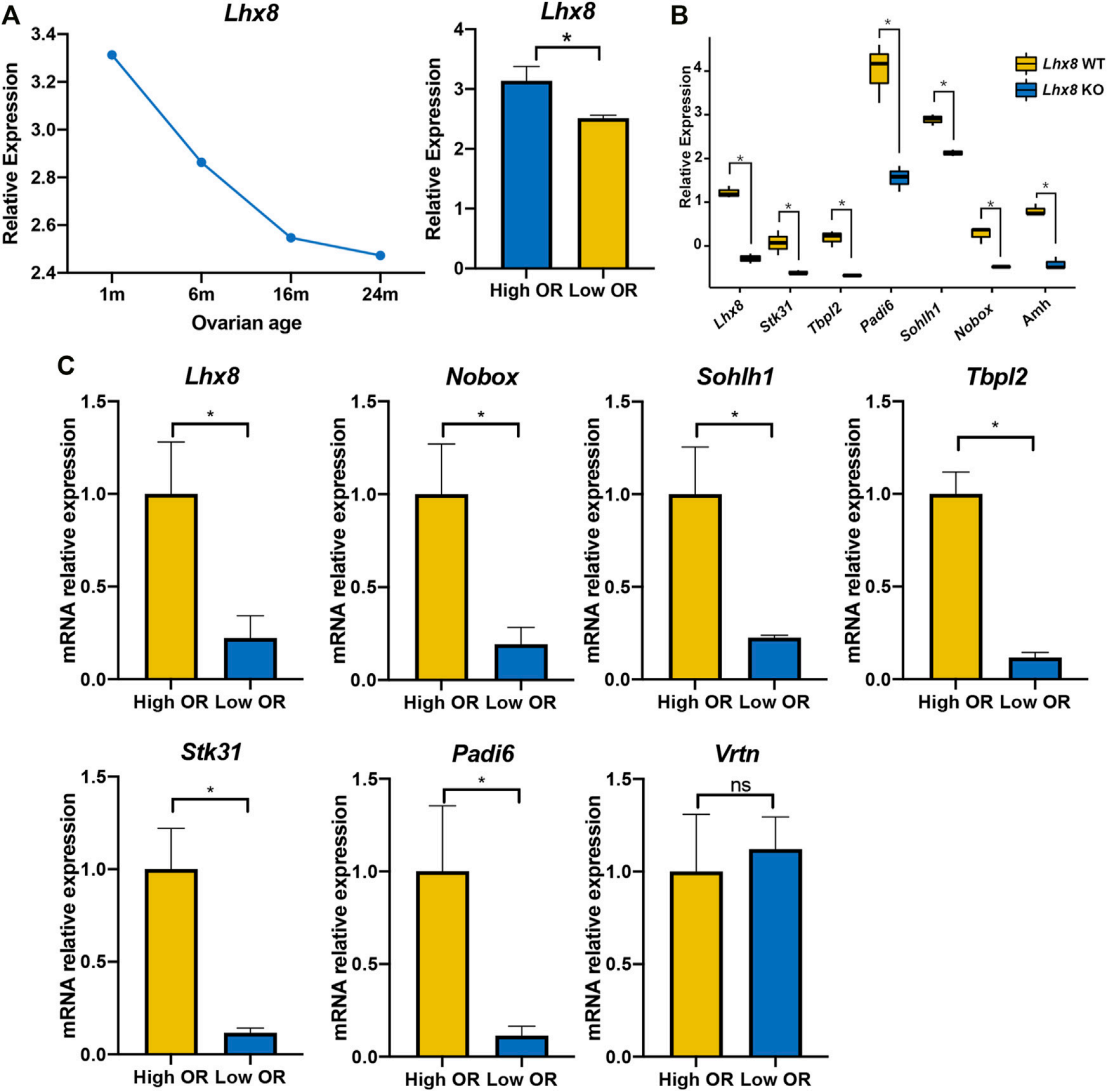
Validation of seven identified genes expression by DOR models. **(A)** The *Lhx8* expression of the entire ovary with different murine BOA from mRNA microarray dataset GSE7502, High OR (1- and 8-month-old group), low OR (16- and 24-month-old group). *, *p* < 0.05. **(B)** The gene expression of the entire ovary in *Lhx8* WT (wild type) and *Lhx8* KO (knockout) from microarray dataset GSE109473; *, *p* < 0.05. **(C)** The genes expression of the entire ovary from our High OR (2-month-old, *n* = 3) and Low OR (8-month-old, *n* = 3) mice. *, *p* < 0.05; ns, *p* > 0.05.

To further investigate the relationship between the expression of these seven genes and OR, we further validated the expression levels of these genes with comparative OR models from the GEO database and our own established. The RNA expression of the entire murine ovary with different murine BOA was obtained by analyzing the RNA microarray dataset GSE7502, and only a probe for *Lhx8* was available for these seven genes mentioned above. The data showed that *Lhx8* expression in mouse ovaries decreased time-dependent and was significantly lower in the low OR group than in younger ovaries (Figure 6A). Then the RNA microarray dataset from *Lhx8* knockout mice was included (GSE109473). It has been reported that *Lhx8* knockout leads to massive depletion of the primordial follicle and induces OR depletion in mice (Ren et al., 2015; D'Ignazio et al., 2018). The results showed that the expression of six genes, including *Lhx8*, *Sohlh1*, *Nobox*, *Stk31*, *Tbpl2*, and *Padi6*, was significantly reduced in ovary tissues from *Lhx8* knockout mice, while no probe of *Vrtn* was observed (Figure 6B).

Moreover, we evaluated the expression of these seven genes in our own established comparative OR models by using quantitative real-time PCR. It showed that six out of the seven genes were expressed at a lower level in low OR ovaries than in young ovaries, while *Vrtn* expression showed no difference (Figure 6C). These data further revealed that the expression of six genes, including *Lhx8*, *Sohlh1*, *Nobox*, *Stk31*, *Tbpl2*, and *Padi6*, was higher in high OR ovaries. And it suggested that these genes expression might be correlated with OR.

## Discussion

The present study identified that seven homologous genes of humans and mice, including *Vrtn*, were highly correlated to OR by combinational analysis of bulk RNA-seq and single-cell RNA-seq data. Further verification consolidated the correlation of six genes with OR based on the published transcriptomic data and quantitative real-time PCR analysis, including *Lhx8*, *Sohlh1*, *Nobox*, *Stk31*, *Tbpl2*, and *Padi6*. Our findings suggested that these six genes might be used as potential biomarkers for evaluating OR at the histological level in mice and humans.

Traditionally, the histological approach to assess the entire ovary’s OR is time-consuming and somewhat statistically subjective by counting the number of primordial follicles in serial H&E/IHC staining sections (Youm et al., 2014; Terren et al., 2019; Mahmoudi Asl et al., 2021). In current clinical practice, the preferred choice for assessing OR is the non-invasive blood detection of serum anti-Müllerian hormone (AMH) (Di Clemente et al., 2021). AMH is secreted by granulosa cells of small growing follicles in the ovary as a potent inhibitor of primordial follicle recruitment. Serum AMH levels strongly correlate with the number of growing follicles, and therefore AMH has received increasing attention as an indirect marker to assess OR (Moolhuijsen and Visser, 2020; Di Clemente et al., 2021). However, the specificity was affected by several factors, such as the age category analyzed and abnormal expression in PCOS (Moolhuijsen and Visser, 2020). A previous study reported that AMH levels increased to plateau at the approximate age of 25 years. Only from this age onward, serum AMH levels start to decline to undetectable levels at menopause, and a negative correlation between serum AMH levels and ages can be observed (Lie Fong et al., 2012). We also observed a similar expression pattern of AMH gene expression with BOA at the histological level (Supplymentary Figure S1), suggesting that it is not appropriate for AMH to represent the natural decline of the primordial follicle pool.

In the present study, we found that the mRNA expression level of six genes was positively correlated with OR in both humans and mice. And the expression of these genes declined linearly along with aging (Figure 5). Additionally, previous studies have documented that *Lhx8*, *Sohlh1*, and *Nobox* have critical roles in regulating primordial follicle activation. Knockout of any of these transcriptional factors causes rapid oocyte loss and ovarian failure (Ren et al., 2015; D'Ignazio et al., 2018; Wang et al., 2020). *Padi6* encodes an enzyme of the peptidyl arginine deiminase family and is uniquely expressed in male and female germ cells. The absence of PADI6 protein results in the dispersal of cytoskeletal sheets in oocytes, which ultimately leads to female infertility (Xiong et al., 2019; Bebbere et al., 2020). Even though *Stk31*, *Tbpl2*, and *Vrtn* are poorly studied in follicle development at the current stage, emerging evidence suggests that these genes are specifically expressed in oocytes and play a crucial role during folliculogenesis (Olesen et al., 2007; Di Pietro et al., 2008; Duan et al., 2018; Yu et al., 2020; Yang et al., 2021). These findings indicated that evaluating OR will be more specific *via* assessing the six gene expression.

Nowadays, growing demand has arisen for objective assessment of OR at the histological level with the development of ART. In the OTC procedure, for example, ovarian tissue from each patient needs to be processed into several fixed-size ovarian tissue slices (e.g., 0.5 cm*0.5 cm*0.1 cm), whereas the evaluation of OR in ovarian tissue slices is not yet standardized (Anderson et al., 2017). The traditional histological assessment of OR is challenging to apply in clinics, while our findings might provide a simple and effective method by detecting these potential biomarkers to evaluate the OR level of ovarian tissue slices routinely collected during OTC without any additional tissue to be collected.

However, several limitations remain in the present study. Firstly, the correlation between the exact primordial follicle numbers and mRNA expression levels needs to be verified. Due to the absence of samples for human DOR in public databases, the expression pattern of these genes and the correlation with OR in human ovarian tissue remains unclear. The sensitivity and specificity of these genes used as biomarkers for OR evaluation need to be validated at histological levels in the large cohort.

In conclusion, the expression level of six genes, including *Lhx8*, *Sohlh1*, *Nobox*, *Stk31*, *Tbpl2*, and *Padi6*, were highly correlated to OR of the primordial follicle pool, suggesting these genes might be used as potential biomarkers for evaluating OR of ovarian tissue both in humans and mice. With the development of ART such as OTC, OTP, growing demand has arisen for objective assessment of OR at the histological level. Our findings might provide a new perspective on clinical assessment of OR and targeted interventions for fertility preservation.

## Data Availability

The original contributions presented in the study are included in the article/Supplementary Material, further inquiries can be directed to the corresponding authors.
