# Supplementary material for: Identification of Biomarkers for Predicting Ovarian Reserve of Primordial Follicle via Transcriptomic Analysis
Source: Front Genet. 2022 May 25;13:879974. doi: 10.3389/fgene.2022.879974 (PMC9174591; doi:10.3389/fgene.2022.879974)
Supplement: Supplementary file 1 [file DataSheet1.DOCX]

Supplementary Material

## Supplementary Figures


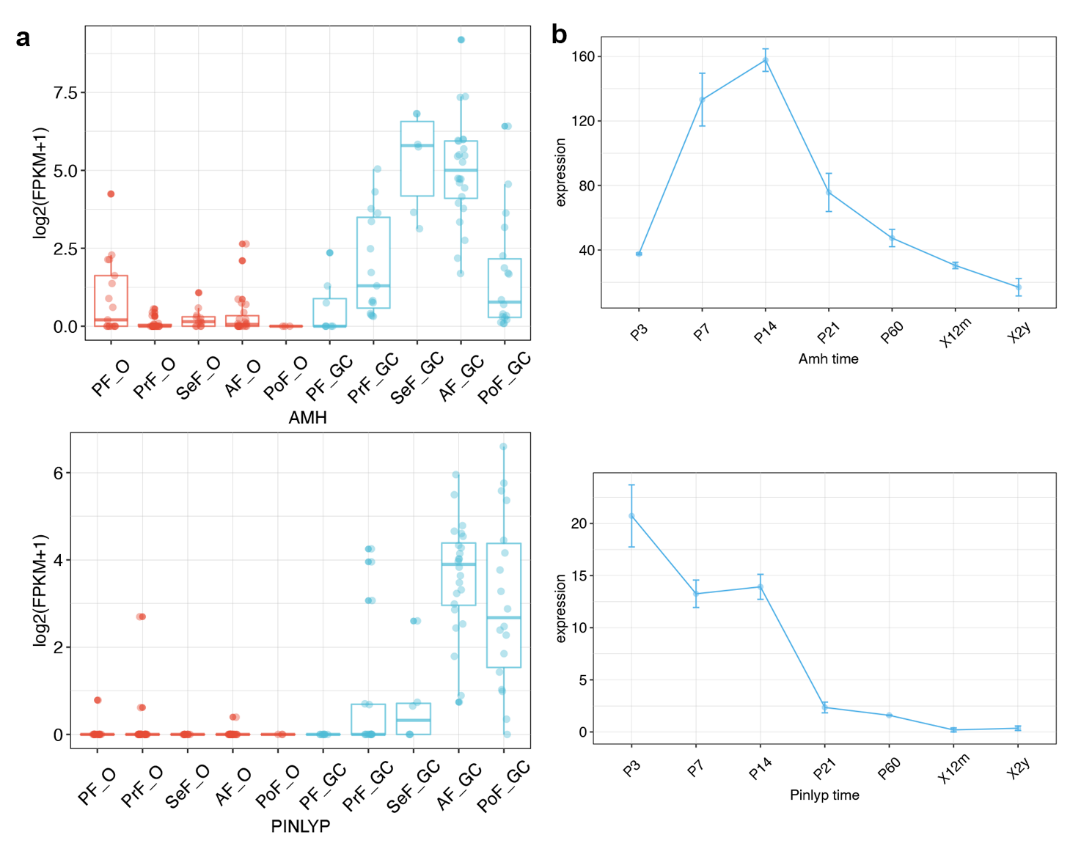


**Supplementary Figure 1.** Expression trends of 2 GC-specific candidate genes in GSE107746 and GSE179888

a. The expression levels of 2 GC-specific candidate genes AMH and PINLYP in the human Oo or GC at five stages of folliculogenesis in the GSE107746 dataset. Oo, oocyte; GC, granulosa cells; PF, primordial follicle; PrF, primary follicle; SeF, secondary follicle; AF, antral follicle; PoF, preovulatory follicle. b. The expression trends of these two genes with BOA changes in murine ovaries from the GSE179888 dataset. P, postnatal day; X12m, postnatal 12 months; X2y, postnatal 2 years.


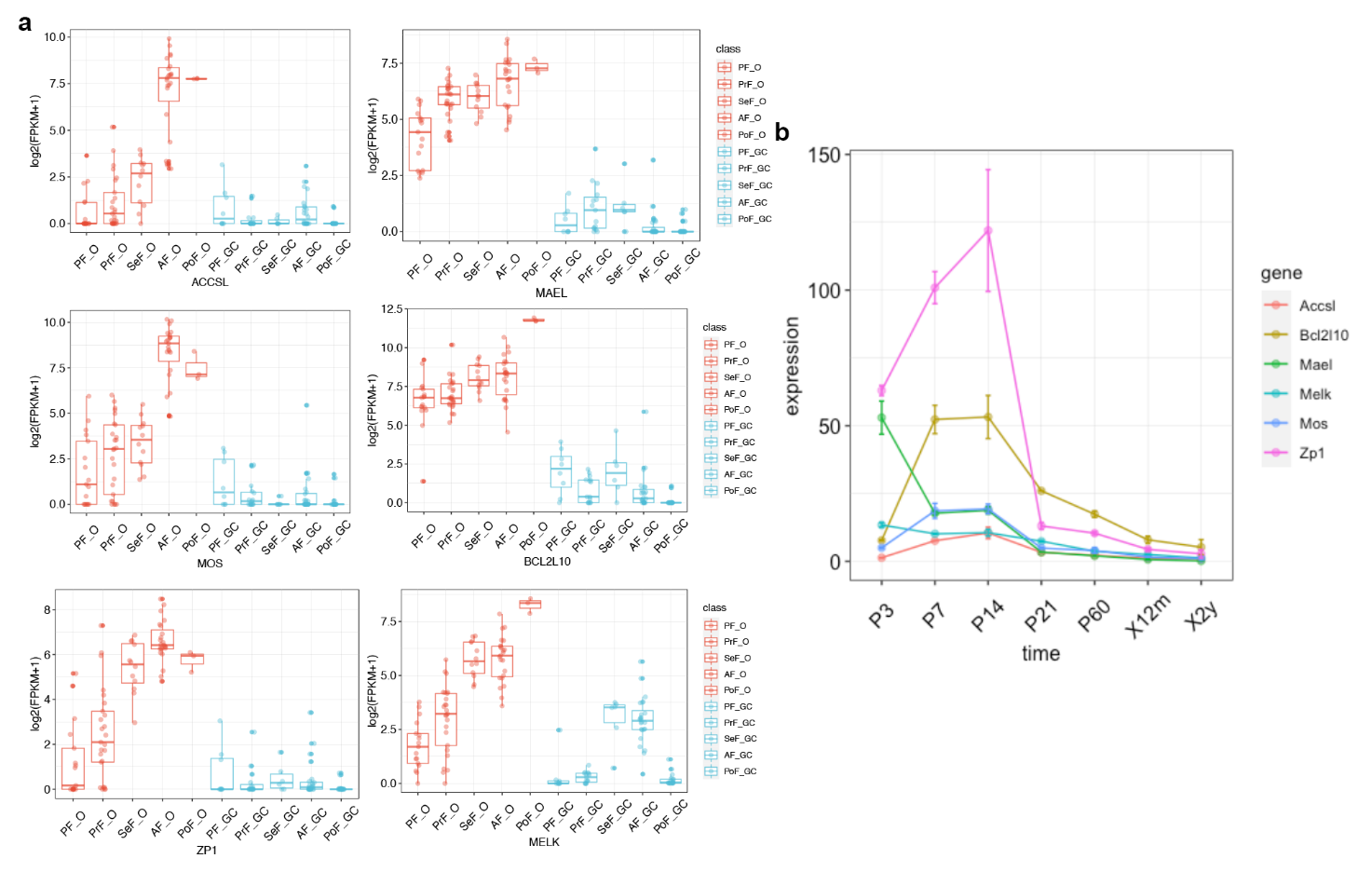


**Supplementary Figure 2.** Expression trends of partial Oo-specific genes positively associated with follicular development

a. The expression levels of some Oo-specific genes positively associated with follicular development in the human Oo or GC at five stages of folliculogenesis in the GSE107746 dataset. Oo, oocyte; GC, granulosa cells; PF, primordial follicle; PrF, primary follicle; SeF, secondary follicle; AF, antral follicle; PoF, preovulatory follicle. b. The expression trends of these genes with BOA changes in murine ovaries from the GSE179888 dataset. P, postnatal day; X12m, postnatal 12 months; X2y, postnatal 2 years.
